# Presenting clinical and imaging features of patients with clinically amyopathic interstitial lung disease associated with myositis-specific autoantibodies

**DOI:** 10.3389/fmed.2024.1392659

**Published:** 2024-04-22

**Authors:** Vasilios Tzilas, Argyrios Tzouvelekis, Vasilina Sotiropoulou, Stylianos Panopoulos, Evangelos Bouros, Eleni Avdoula, Jay H. Ryu, Demosthenes Bouros

**Affiliations:** ^1^5th Respiratory Department, Chest Diseases Hospital “Sotiria”, Athens, Greece; ^2^Department of Respiratory Medicine, Medical School, University of Patras, Patras, Greece; ^3^1st Department of Propaedeutic and Internal Medicine, and Joint Rheumatology Program, National and Kapodistrian University of Athens Medical School, Athens, Greece; ^4^Athens Medical Center, Athens, Greece; ^5^Division of Pulmonary and Critical Care Medicine, Mayo Clinic, Rochester, MN, United States; ^6^1st Department of Respiratory Medicine, Medical School, National Kapodistrian University of Athens, and Athens Medical Center, Athens, Greece

**Keywords:** myositis, amyopathic, non specific interstitial pneumonia, organizing pneumonia, HRCT, idiopathic inflammatory myopathies, antisynthetase syndrome

## Abstract

**Background:**

Lung involvement in the context of idiopathic inflammatory myopathies has significant impact on outcome; early and accurate diagnosis is important but can be difficult to achieve. In particular, patients without clinically evident muscle involvement pose a significant diagnostic challenge.

**Methods:**

A computer-assisted search was conducted to identify patients with amyopathic interstitial lung disease associated with the presence of myositis-specific autoantibodies. Medical records and chest imaging studies were reviewed to identify clinical and radiologic features at presentation.

**Results:**

Of the 35 patients with amyopathic interstitial lung disease associated with myositis-specific autoantibodies, the median age was 65 years (range 43–78) and 20 were women (57%). Of the patients, 34% had previously visited the rheumatology department. Presenting symptoms consisted of dyspnea (94%), cough (43%), and arthritis (23%). Raynaud phenomenon, “mechanic hands,” Gottron papules, and inspiratory crackles were present in 23, 31, 9, and 74% of patients, respectively. After a detailed history, none of the patients reported muscle weakness, while four (11%) exhibited increased CK levels; of these four, two had a concomitant increase in aldolase levels. Median FVC was 79% predicted (range: 49–135) and median DLco was 50% predicted (range: 17–103). HRCT pattern was suggestive of an alternative to UIP pattern in 31/33 (94%) patients; the most common imaging patterns were NSIP (49%) and NSIP/OP (39%).

**Conclusion:**

In patients with NSIP and NSIP/OP pattern, the presence of amyopathic interstitial lung disease associated with myositis-specific autoantibodies should be considered even in the absence of clinical evident myositis.

## Introduction

The idiopathic inflammatory myopathies (IIM) represent a heterogeneous group of autoimmune diseases that are characterized by chronic inflammation of skeletal muscle leading to muscle weakness (1, 2). Our understanding regarding the spectrum and complexity of IIM has expanded significantly in recent years. The availability of comprehensive myositis panels helped us understand the complex nature of IIM, which are now conceptualized as systemic inflammatory diseases with multiple organ involvement. Furthermore, in certain IIM subtypes, lung involvement in the form of interstitial lung disease (ILD) prevails in terms of both clinical manifestation and prognosis (3, 4). Antisynthetase syndrome (ASyS) is a characteristic example. ILD is seen in 70–95% of patients with ASyS (5) and is a significant driver of morbidity and mortality across IIMs (6, 7). Available data on patients with ILD associated with amyopathic dermatomyositis are scarce (8, 9).

Despite the progress that has been achieved, the importance of ILD in patients with IIM is underappreciated; it is still not considered a defining clinical feature in the most recent classification criteria for IIM by the European Alliance of Associations for Rheumatology and the American College of Rheumatology (EULAR/ACR) (10). The failure to accurately diagnose and institute appropriate immunomodulatory treatment can have detrimental effects on patients’ outcomes. At the same time, immunomodulation in the setting of an infectious acute exacerbation of ILD can be devastating. Thus, timely and accurate diagnosis can be challenging considering the rarity but also lethality of IIM-associated ILD (11). The main reasons for this difficulty are that ILD can be the initial and sometimes only presenting manifestation in up to 40% of patients with myositis (12), and muscle involvement can be subclinical with no laboratory evidence of muscle inflammation.

With this study, we report the presenting clinical and imaging features of patients with clinically amyopathic ILD associated with myositis-specific autoantibodies, aiming to facilitate recognition of amyopathic ILD.

## Methods

A computer-assisted search was conducted to identify patients with clinically amyopathic ILD associated with myositis-specific antibodies seen at the Department of Respiratory Medicine, University Hospital of Patras, Patras, Greece; the 5th Department of Pneumonology, General Hospital for Thoracic Diseases Sotiria, Athens, Greece; and Athens Medical Center, Athens, Greece. Data collection and analysis were approved by the Institutional Review Board and the Local Ethics Committee (protocol number 28746/9-12-2019). Antibody profile was obtained using the EUROLINE Autoimmune Inflammatory Myopathies 16 Ag [IgG] test kit, which provides a qualitative assay for human autoantibodies of the immunoglobulin class IgG to 16 different antigens in serum or plasma: Mi-2α, Mi-2β, TIF1γ, MDA5, NXP2, SAE1, Ku, PM-Scl100, PM-Scl75, Jo-1, SRP, PL-7, PL-12, EJ, OJ, and Ro-52.

Medical records and chest imaging studies were reviewed to identify clinical, serological, and radiologic features along with functional indices including forced vital capacity % predicted (FVC% pred). diffusing capacity of the lung for carbon monoxide % predicted (DLCO% pred), and cellular profiles of bronchoalveolar lavage (BAL) fluid. We extracted demographic and clinical data including age, sex, age at diagnosis, smoking status, presenting respiratory and systemic symptoms and signs, and levels of creatine kinase (CK) and aldolase at presentation.

We excluded patients with solely myositis-associated autoantibodies such as PM-Scl-75/100, Ku, and Ro-52, as they are not specific for IIM and can be found in other connective tissue diseases (CTDs) including systemic sclerosis and systemic lupus erythematosus (13–15).

The distribution of parenchymal abnormalities on CT was examined in the axial (predominantly peripheral, central, or diffuse) and craniocaudal planes (predominantly cranial, basal, or diffuse) (16, 17). Furthermore, we assessed if the distribution was peribronchial and whether there was subpleural sparing or “hugging” of the diaphragms. Subpleural sparing was defined as involvement of the peripheral lung but with relative sparing of the immediate subpleural lung parenchyma. Hugging of the diaphragms was defined as extreme basilar predominance of findings, including reticular and ground-glass opacities and traction bronchiectasis with or without consolidation, that hug or “pancake” the diaphragms (18). The straight edge sign was characterized by fibrotic changes isolated to the lung bases without substantial extension along the lateral margins of the lungs on coronal images (19). The predominant imaging pattern of abnormality was classified as usual interstitial pneumonia (UIP), probable UIP, indeterminate for UIP, or suggestive of an alternative diagnosis pattern consistent with the latest ATS/ERS/JRS/ALAT guidelines for idiopathic pulmonary fibrosis (IPF) (20). Nonspecific interstitial pneumonia (NSIP) pattern was recognized by the presence of predominant ground-glass opacities with a predominantly basal distribution with or without reticulation or traction bronchiectasis and no or only minimal honeycombing as described by Silva et al. (16). Organizing pneumonia (OP) pattern consisted of bilateral areas of consolidative opacities, while consolidations superimposed on a background of ground-glass opacities, with or without reticulations or traction bronchiectasis, was classified as NSIP/OP pattern (18).

## Results

We identified 35 patients with amyopathic ILD associated with the presence of myositis-specific autoantibodies; 57% were women, 63% were never smokers, and the median age was 65 years (range 43–78). All patients were diagnosed at the outpatient clinic, and none were in need of supplemental oxygen. Aminoacyl-tRNA synthetases antibodies were identified in 28 patients (80%); the majority were anti-Jo-1 positive (15/28, 54%), while the remaining patients were positive for anti-PL-7 (25%), anti-PL-12 (11%), anti-EJ (7%), and anti-OJ (4%). The remaining patients exhibited anti-Mi-2b (8%), anti-MDA5 (6%), anti-Mi2a (3%), and anti-NXP2 (3%). Concomitant anti-Ro-52 antibodies were seen in 16 (46%) patients. A titer of antinuclear antibodies (ANA) greater than 1:160 was observed in nine (26%) patients (Table 1).

**Table 1:** Demographic and clinical parameters.

Characteristic	Value
Sex, no. (%)	
Female	20 (57)
Age, years, median (range)	65 (43–78)
Smoking status, no. (%)	
Never smoker Ex smoker Current smoker	22 (63) 11 (31) 2 (6)
Presenting symptoms, no. (%)	
Dyspnea	33 (94)
Cough	15 (43)
Chest pain	0 (0)
Signs, no. (%)	
Crackles	26 (74)
Raynaud phenomenon	8 (23)
Mechanic hands	11 (31)
Gottron papules	3 (9)
Positive aminoacyl-tRNA synthetases antibodies Anti-Jo-1 Anti-PL-7 Anti-PL-12 Anti EJ Anti OJ	28 (80%) 15/28 (54) 7 (25) 3 (11) 2 (7) 1 (3)
Anti Mi-2a Anti-Mi2b Anti-MDA5 Anti-NXP2	1 (3) 3 (8) 2 (6) 1 (3)
Anti-Ro-52	16 (46)
Elevated CPK levels	4 (11)
Elevated aldolase levels	2 (6)
FVC% predicted, median (range)	79 (49–135)
DLco % predicted, median (range)	51 (17–103)

CPK, creatine phosphokinase; FVC: forced vital capacity; DLco, diffusing capacity of the lung for carbon monoxide.

The most common presenting symptoms consisted of dyspnea (94%), cough (43%), and arthritis (23%). None of the patients reported muscle weakness or muscle pain. On clinical examination, the most common findings were inspiratory crackles (74%), Raynaud phenomenon (23%), “mechanic hands” (31%), and Gottron papules (9%). Elevated CK level was observed in four (11%) patients, of whom two had a concomitant increase in aldolase levels. Of the patients, 34% had previously visited the rheumatology department. Exposure to an inciting antigen known to cause hypersensitivity pneumonitis was reported by 13 (37%) patients.

Median FVC % predicted was 79% (range: 49–135) and median DLco % predicted was 50.5% (range: 17–103). At presentation, a history of malignancy was reported by four (11%) patients: bronchogenic lung cancer in two patients, colon cancer in one patient, and uterine cancer in one patient.

BAL was performed in 17 (49%) patients. BAL lymphocytosis ≥20% was seen in 65% and ≥ 30% in 35% of cases; median percentage of BAL lymphocytes was 24% (range 1–50%). Median CD4/CD8 ratio (available in eight patients with BAL lymphocytosis ≥20%) was 0.38 (range: 0.1–0.96).

Baseline HRCT was available for 33 patients (Table 2). HRCT pattern was suggestive of an alternative diagnosis to UIP in 31 (94%) patients. The most common imaging patterns were NSIP (49%) and NSIP/OP (39%) followed by OP (6%), probable UIP (3%), and indeterminate for UIP (3%).

**Table 2:** Chest computed tomography findings at presentation.

Parameter	*n* (%)
**Distribution**	
**Craniocaudal**	
Upper/mid Lower Diffuse	1 (3) 25 (76) 7(21)
**Axial**	
Peripheral Central Diffuse Subpleural sparing Diaphragmatic hugging Straight edge sign Perilobular Peribronchial	21(64) 1 (3) 11 (33) 18 (55) 13 (36) 3 (9) 11 (33) 14 (42)
**Morphology**	
GGO Consolidation Reticulation Traction bronchiectasis Honeycombing Reversed halo sign	32 (97) 15 (45) 19 (58) 24 (73) 0 (0) 0 (0)
**Pattern**	
NSIP NSIP/OP OP Probable UIP Indeterminate for UIP	16 (49) 13 (39) 2 (6) 1 (3) 1 (3)
Pleural effusion Pericardial effusion	1 (3) 1 (3)

HRCT, high resolution computed tomography; UIP, usual interstitial pneumonia, NSIP, non-specific interstitial pneumonia, OP, organizing pneumonia; GGO, ground glass opacities.

**Figure 1:**
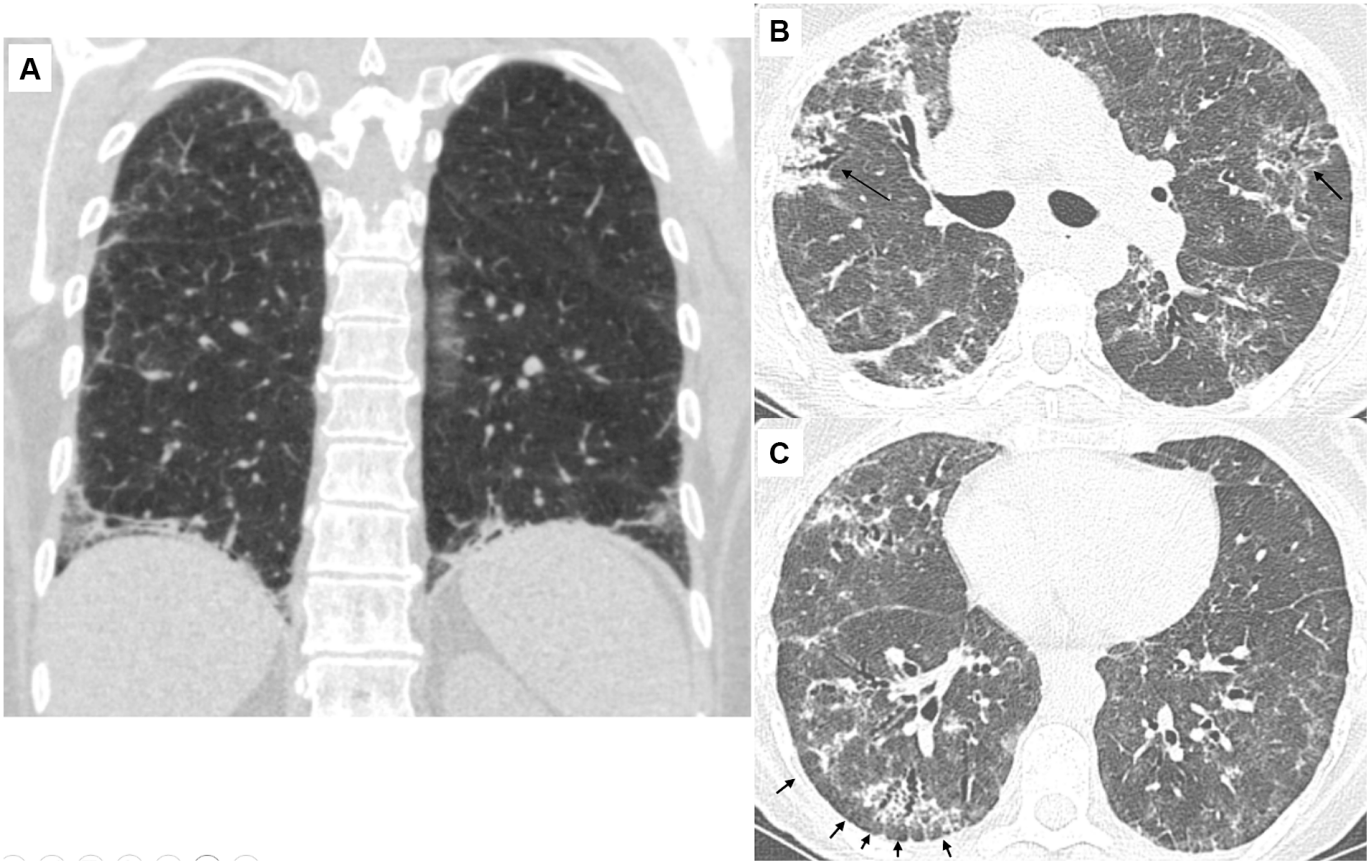
Characteristic CT findings of the lungs in patients with antisynthetase syndrome. **(A)** 56-year-old female (anti-Jo-1 positive). Coronal view. There is reticulation with ground-glass opacities and traction bronchiectasis. Note the extreme bibasilar location of the abnormal findings that seem to “hug” or “pancake” the diaphragms. This finding is considered highly suggestive of underlying polymyositis/dermatomyositis, especially antisynthetase syndrome. **(B)** 66-year-old female (anti-PL-7 positive). There are diffuse ground-glass opacities resulting in a mosaic pattern. There are also consolidative areas centered around bronchi (arrows) that have the characteristic corkscrew appearance in line with traction bronchiectasis. The presence of mosaic attenuation and bronchocentricity can erroneously support a diagnosis of hypersensitivity pneumonitis, especially in patients with a history of exposure to an inciting antigen. **(C)** 66-year-old female (anti-PL-7 positive). There are diffuse ground-glass opacities and traction bronchiectasis with the characteristic cork-screw appearance. Note that the fibrotic changes are located in the periphery of the lungs with relative sparing of the immediate subpleural parenchyma. The subpleural sparing is subtle in the left lung but evident in the right lung (arrows).

Fibrotic changes, based on the presence of traction bronchiectasis, were observed in 24 (73%) patients. The predominant distribution of parenchymal abnormalities was lower lung (76%) in the craniocaudal plane and peripheral (64%) in the axial plane. Subpleural sparing was present in 18 (55%) patients, diaphragmatic hugging in 13 (36%) patients, and the straight edge sign in three (9%) patients. All patients with diaphragmatic hugging were positive for anti-aminoacyl t-RNA synthetases antibodies except one who manifested anti-MDA5 antibodies (Figure 1).

Surgical lung biopsy was performed on four (11%) patients: in two cases it was interpreted as compatible with hypersensitivity pneumonitis, in one case as UIP pattern, and in the remaining case as NSIP pattern with suspicion of underlying CTD.

## Discussion

In this retrospective study, we studied the presenting clinical and imaging features of patients with clinically amyopathic ILD associated with myositis-specific autoantibodies. Characteristic signs of IIM such as “mechanic hands” and Gottron papules were found only in a minority of patients. Thus, in the context of unexplained ILD, the absence of these findings does not exclude the diagnosis of underlying IIM.

From an imaging perspective, in the vast majority of cases (88%) the encountered CT pattern was NSIP and NSIP/OP. OP as a standalone pattern was seen only in 6%. Honeycombing was not seen in any patient, while only one patient exhibited probable UIP pattern. Our results agree with prior studies on the chest imaging findings of ILD associated with IIM that were not limited to amyopathic cases (21–23). For example, CT scan did not reveal honeycombing in 70 patients in one of the earliest studies of ILDs in the context of IIM (24). Misra et al. studied 36 patients with ILD associated with myositis-specific antibodies, and none of the patients manifested honeycombing on chest CT scans (22). Debray et al. (17) described the imaging findings of 33 patients with ASyS and none of the patients had a UIP pattern at presentation; the main pattern was NSIP and/or NSIP/OP, which were present in 69% of cases. They reported a similar rate of traction bronchiectasis at 70% (73% in our study). In another study comprising 64 patients with ASyS, the main CT pattern was NSIP and/or fibrotic OP, being present in 89% of cases, while OP without fibrosis was seen in 6% of cases, consistent with our findings (21). NSIP and/or NSIP/OP was also the most frequent imaging pattern in a large cohort of 103 anti-jo-1 positive patients, being observed in 74% of cases (25). We found subpleural sparing in 55% of our cases, slightly higher than what has been reported so far (33–40%) (17, 21).

Fisher et al. were the first to describe hugging or “pancaking” of the diaphragm in patients with ASyS (18). According to their experience, this sign is considered fairly specific to ASyS-related ILD. Despite its diagnostic accuracy, this radiographic sign has not been extensively studied. In our cohort, hugging of the diaphragm was present in about one-third of patients, almost exclusively seen in patients with ASyS. Diaphragmatic hugging should raise suspicion of underlying IIM and especially ASyS. However, further studies are needed to prove its diagnostic accuracy while its absence should not preclude the diagnosis of IIM-associated ILD.

In the 2018 IPF guidelines by ATS/ERS/JRS/ALAT, most panelists agreed with serologic testing with myositis panel in the evaluation of patients with suspected IPF. However, it seems that ILD in the context of IIM rarely presents with UIP or probable UIP. According to our findings, the clinical need to perform extensive serologic testing, including myositis panel, mainly applies to patients with NSIP and/or NSIP/OP even in the absence of clinical and or laboratory evidence of myositis. This was highlighted more than a decade ago by Fisher et al. (18). They evaluated 37 patients labeled as “idiopathic” ILD with negative ANA and anti-jo-1 antibodies and identified nine cases with anti-PL-7 or anti-PL-12-associated ASyS. Likewise, Fidler et al. (26) retrospectively evaluated 165 patients diagnosed with idiopathic ILD. Myositis-associated or specific antibodies were detected in 26.7% of patients.

Exposure to an inciting antigen known to cause hypersensitivity pneumonitis was reported by about one-third of patients in our cohort. This finding represents an important attribute of our study and may have diagnostic implications as it can lead to an erroneous diagnosis of hypersensitivity pneumonitis. This is not an uncommon scenario; we have previously reported four patients with ASyS masquerading as hypersensitivity pneumonitis (27). It has been suggested that environmental triggers may play a significant role in the pathogenesis of IIM by causing chronic immune activation in genetically predisposed individuals. Occupational, domiciliary, and environmental exposure has been reported in 50 to 59% of patients with IIM (28, 29). In patients with IIM and primarily pulmonary involvement, bird and feather-containing pillow exposure was present in 10 out of 23 patients (43%) (28). The term hypersensitivity pneumonitis with autoimmune features (HPAF) has been introduced for HP patients with evidence of a concurrent defined autoimmune disease or autoimmune features suggestive of CTD (30). However, caution is needed as HPAF is not a clinical term and may obscure the actual diagnosis. Anchoring to a false diagnosis of HP (and/or HPAF) may hamper recognition of underlying IIM and delay timely application of immunomodulatory therapies, leading to detrimental clinical outcomes.

“Interstitial pneumonia with autoimmune features” (IPAF) was proposed as an entity for research purposes to reduce heterogeneity in studies of patients with ILD and features of autoimmune disease but without manifestations to meet the diagnosis of a specific CTD (31). Although IPAF was not meant to be a diagnosis, it is commonly used as such in clinical practice, creating confusion. Classification criteria for this entity includes clinical, serologic, and morphologic domains. Notably, the serological domain includes the aminoacyl-tRNA synthetases and anti-MDA autoantibodies, while the imaging domain includes NSIP and NSIP/OP patterns. Thus, such patients with no extrapulmonary manifestations, as included in our study, could potentially be classified as having IPAF. However, IPAF designation would underestimate the high specificity of the myositis-specific autoantibodies which, in the appropriate clinico-radiological context, allows recognition of certain phenotypes of IIM.

BAL lymphocytosis ≥20% and ≥ 30% was seen in two-thirds and one-third of cases, respectively. The diagnostic significance of BAL lymphocytosis should be interpreted with caution and in consideration of the clinico-radiological context. BAL lymphocytosis can be helpful by revealing diagnoses other than IPF (32). However, neither the presence nor the degree of BAL lymphocytosis can point to a specific diagnosis (33). On the contrary, it can be misleading and enhance anchoring bias. For example, in the presence of an inciting antigen, the finding of BAL lymphocytosis could be considered as further evidence to support the diagnosis of hypersensitivity pneumonitis.

In our study, ANA titer greater or equal to 1:320 was seen in just one-quarter of cases. This is in line with previous observations that a negative ANA test does not reliably indicate the absence of circulating autoantibodies such as myositis-specific autoantibodies and should not be considered as definitively excluding an underlying CTD (10, 34, 35). Furthermore, testing only for anti-jo-1 antibodies is not sufficient as it can miss a significant number of patients with IIM. In our cohort, myositis-specific antibodies not related to ASyS were present in 20%. Regarding patients with ASyS, 46% manifested non-jo-1 aminoacyl-tRNA synthetases antibodies. Thus, simply relying only on anti-jo-1 testing, about two-thirds of our patients would have remained undiagnosed. In a large study including 828 patients with ASyS, isolated ILD at presentation was present in 154 (19%) of cases. Of these patients, only 14 (9%) were anti-jo-1 positive (36).

Interestingly, one-third of patients reported a prior visit to rheumatologists. It should be emphasized that pulmonologists are likely to see a different phenotype of IIM patients compared to rheumatologists, as systemic manifestations are lacking. Indeed, it has been shown that patients with ASyS presenting initially to pulmonologists had significantly fewer musculoskeletal symptoms and/or biochemical evidence of myositis compared with those presenting to rheumatologists (35).

Surgical lung biopsy was performed in four cases but only in one was it suggestive of underlying CTD. Notably, in two cases it was interpreted as suggestive of HP and would correspond to a probable histopathologic HP (37). It must be emphasized that the pathological separation between fibrotic (chronic) HP and fibrotic CTD-ILD can be extremely challenging as there are overlapping features and there is no single pathologic finding (e.g., giant cells/granulomas or germinal centers) that clearly distinguishes them (38). For example, giant cells/granulomas were found in 56% of HP patients but were also present in one-third of CTD-ILD patients; peribronchial fibrosis was present in 81% of HP patients but also in 58% of CTD-ILD patients (38).

Our study has limitations due to its retrospective nature. Also, the sample size is relatively modest. However, IIM-related ILD is relatively rare, and we further narrowed our study cohort to amyopathic patients, who are more difficult to diagnose. We excluded patients with solely myositis-associated autoantibodies and restricted our study to patients with myositis-specific antibodies. Finally, our study was not designed to provide long-term data regarding follow up and treatment response. Our goal was to describe the clinical and imaging features to raise awareness for this rare disease and highlight that the lungs can be the predominant site of involvement in patients with IIM.

The increasing availability of comprehensive myositis panels demonstrated that autoimmune-induced interstitial lung injuries represent frequent manifestations of IIM in the absence of clinical evident myositis. It has clinical implications since, in certain contexts, the presence of myositis-specific autoantibodies can help establish a diagnosis without tissue confirmation (3). Current nomenclature and the concept of IPAF underestimates the importance of pulmonary involvement in the field of ΙΙΜ. ILD is absent in the recent classification criteria for IIM formulated by the EULAR/ACR. It is important to narrow the existing gap between pulmonologists and rheumatologists and acknowledge the lungs as a major site of involvement in certain phenotypes of IIM (3, 4). Routine inclusion of myositis panel in serologic testing for CTD seems warranted in the diagnostic evaluation of patients presenting with an NSIP and/or NSIP/OP imaging pattern, including those with suspected fibrotic (chronic) HP.

## Data availability statement

The raw data supporting the conclusions of this article will be made available by the authors, without undue reservation.

## Ethics statement

The studies involving humans were approved by the University of Patras, Medical School. The studies were conducted in accordance with the local legislation and institutional requirements. Written informed consent for participation was not required from the participants or the participants’ legal guardians/next of kin in accordance with the national legislation and institutional requirements.

## Author contributions

VT: Conceptualization, Formal analysis, Investigation, Methodology, Project administration, Writing – review & editing, Data curation. AT: Conceptualization, Data curation, Formal analysis, Investigation, Methodology, Project administration, Writing – original draft, Writing – review & editing. VS: Data curation, Formal analysis, Investigation, Writing – review & editing. SP: Conceptualization, Data curation, Formal analysis, Investigation, Writing – review & editing. EB: Data curation, Formal analysis, Investigation, Writing – review & editing. EA: Data curation, Formal analysis, Writing – review & editing. JR: Conceptualization, Formal analysis, Investigation, Methodology, Supervision, Writing – review & editing. DB: Conceptualization, Formal analysis, Investigation, Methodology, Project administration, Supervision, Writing – review & editing.

## References

[1] Dalakas MC. Inflammatory muscle diseases. N Engl J Med. (2015) 372:1734–47. doi: 10.1056/NEJMra1402225 25923553

[2] Lundberg IE de Visser M Werth VP. Classification of myositis. Nat Rev Rheumatol. (2018) 14:269–78. doi: 10.1038/nrrheum.2018.41 29651121

[3] Mariampillai K Granger B Amelin D Guiguet M Hachulla E Maurier F. Development of a new classification system for idiopathic inflammatory myopathies based on clinical manifestations and myositis-specific autoantibodies. JAMA Neurol. (2018) 75:1528–37. doi: 10.1001/jamaneurol.2018.2598, PMID: 30208379 PMC6583199

[4] Hallowell RW Danoff SK. Diagnosis and Management of Myositis-Associated Lung Disease. Chest. (2023) 163:1476–91. doi: 10.1016/j.chest.2023.01.031 36764512

[5] Shappley C Paik JJ Saketkoo LA. Myositis-related interstitial lung diseases: diagnostic features, treatment, and complications. Curr Treat Options Rheumatol. (2019) 5:56–83. doi: 10.1007/s40674-018-0110-6, PMID: 31984206 PMC6980290

[6] Aggarwal R Cassidy E Fertig N Koontz DC Lucas M Ascherman DP. Patients with non-Jo-1 anti-tRNA-synthetase autoantibodies have worse survival than Jo-1 positive patients. Ann Rheum Dis. (2014) 73:227–32. doi: 10.1136/annrheumdis-2012-201800, PMID: 23422076 PMC4031026

[7] Johnson C Pinal-Fernandez I Parikh R Paik J Albayda J Mammen AL. Assessment of mortality in autoimmune myositis with and without associated interstitial lung disease. Lung. (2016) 194:733–7. doi: 10.1007/s00408-016-9896-x 27166633 PMC11678787

[8] Cottin V Thivolet-Béjui F Reynaud-Gaubert M Cadranel J Delaval P Ternamian PJ. Interstitial lung disease in amyopathic dermatomyositis, dermatomyositis and polymyositis. Eur Respir J. (2003) 22:245–50. doi: 10.1183/09031936.03.00026703 12952255

[9] Suda T Fujisawa T Enomoto N Nakamura Y Inui N Naito T. Interstitial lung diseases associated with amyopathic dermatomyositis. Eur Respir J. (2006) 28:1005–12. doi: 10.1183/09031936.06.00038806 16837503

[10] Aggarwal R Dhillon N Fertig N Koontz D Qi Z Oddis CV. A negative antinuclear antibody does not indicate autoantibody negativity in myositis: role of Anticytoplasmic antibody as a screening test for Antisynthetase syndrome. J Rheumatol. (2017) 44:223–9. doi: 10.3899/jrheum.160618, PMID: 27909085

[11] Tzilas V Ryu JH Sfikakis PP Tzouvelekis A Bouros D. Antisynthetase syndrome with predominant lung involvement. An Easy to miss diagnosis. Pulmonology. (2023) 29:271–2. doi: 10.1016/j.pulmoe.2023.02.009, PMID: 36906463

[12] Koreeda Y Higashimoto I Yamamoto M Takahashi M Kaji K Fujimoto M. Clinical and pathological findings of interstitial lung disease patients with anti-aminoacyl-tRNA synthetase autoantibodies. Int Med. (2010) 49:361–9. doi: 10.2169/internalmedicine.49.2889, PMID: 20190466

[13] McHugh NJ Tansley SL. Autoantibodies in myositis. Nat Rev Rheumatol. (2018) 14:290–302. doi: 10.1038/nrrheum.2018.56 29674612

[14] Tzouvelekis A Karampitsakos T Bouros E Tzilas V Liossis SN Bouros D. Autoimmune biomarkers, antibodies, and immunologic evaluation of the patient with fibrotic lung disease. Clin Chest Med. (2019) 40:679–91. doi: 10.1016/j.ccm.2019.06.002, PMID: 31376900

[15] Ashton C Paramalingam S Stevenson B Brusch A Needham M. Idiopathic inflammatory myopathies: a review. Intern Med J. (2021) 51:845–52. doi: 10.1111/imj.15358 34155760

[16] Silva CI Müller NL Hansell DM Lee KS Nicholson AG Wells AU. Nonspecific interstitial pneumonia and idiopathic pulmonary fibrosis: changes in pattern and distribution of disease over time. Radiology. (2008) 247:251–9. doi: 10.1148/radiol.2471070369, PMID: 18270375

[17] Debray MP Borie R Revel MP Naccache JM Khalil A Toper C. Interstitial lung disease in anti-synthetase syndrome: initial and follow-up CT findings. Eur J Radiol. (2015) 84:516–23. doi: 10.1016/j.ejrad.2014.11.026, PMID: 25541020

[18] Fischer A Swigris JJ du Bois RM Lynch DA Downey GP Cosgrove GP. Anti-synthetase syndrome in ANA and anti-Jo-1 negative patients presenting with idiopathic interstitial pneumonia. Respir Med. (2009) 103:1719–24. doi: 10.1016/j.rmed.2009.05.001, PMID: 19497723 PMC2857337

[19] Chung JH Cox CW Montner SM Adegunsoye A Oldham JM Husain AN. CT features of the usual interstitial pneumonia pattern: differentiating connective tissue disease-associated interstitial lung disease from idiopathic pulmonary fibrosis. AJR Am J Roentgenol. (2018) 210:307–13. doi: 10.2214/AJR.17.18384, PMID: 29140119 PMC12525794

[20] Raghu G Remy-Jardin M Richeldi L Thomson CC Inoue Y Johkoh T. Idiopathic pulmonary fibrosis (an update) and progressive pulmonary fibrosis in adults: an official ATS/ERS/JRS/ALAT clinical practice guideline. Am J Respir Crit Care Med. (2022) 205:e18–47. doi: 10.1164/rccm.202202-0399ST, PMID: 35486072 PMC9851481

[21] Waseda Y Johkoh T Egashira R Sumikawa H Saeki K Watanabe S. Antisynthetase syndrome: pulmonary computed tomography findings of adult patients with antibodies to aminoacyl-tRNA synthetases. Eur J Radiol. (2016) 85:1421–6. doi: 10.1016/j.ejrad.2016.05.012, PMID: 27423682

[22] Misra AK Wong NL Healey TT Lally EV Shea BS. Interstitial lung disease is a dominant feature in patients with circulating myositis-specific antibodies. BMC Pulm Med. (2021) 21:370. doi: 10.1186/s12890-021-01737-7, PMID: 34775966 PMC8591876

[23] Karampitsakos T Tzilas V Papaioannou O Chrysikos S Vasarmidi E Juge PA. Clinical features and outcomes of patients with myositis associated-interstitial lung disease. Front Med. (2023) 9:1096203. doi: 10.3389/fmed.2022.1096203, PMID: 36698813 PMC9868310

[24] Douglas WW Tazelaar HD Hartman TE Hartman RP Decker PA Schroeder DR. Polymyositis–dermatomyositis-associated interstitial lung disease. Am J Respir Crit Care Med. (2001) 164:1182–5. doi: 10.1164/ajrccm.164.7.2103110, PMID: 11673206

[25] Zamora AC Hoskote SS Abascal-Bolado B White D Cox CW Ryu JH. Clinical features and outcomes of interstitial lung disease in anti-Jo-1 positive antisynthetase syndrome. Respir Med. (2016) 118:39–45. doi: 10.1016/j.rmed.2016.07.009, PMID: 27578469

[26] Fidler L Doubelt I Kandel S Fisher JH Mittoo S Shapera S. Screening for myositis antibodies in idiopathic interstitial lung disease. Lung. (2019) 197:277–84. doi: 10.1007/s00408-019-00212-9 30838434

[27] Tzilas V Sfikakis PP Bouros D. Antisynthetase syndrome masquerading as hypersensitivity pneumonitis. Respiration. (2021) 100:1105–13. doi: 10.1159/000516508, PMID: 34148050

[28] Costa AN Kawano-Dourado L Shinjo SK Carvalho CR Kairalla RA. Environmental exposure in inflammatory myositis. Clin Rheumatol. (2014) 33:1689–90. doi: 10.1007/s10067-014-2567-5, PMID: 24651912

[29] Labirua-Iturburu A Selva-O’Callaghan A Zock JP Orriols R Martínez-Gómez X Vilardell-Tarrés M. Occupational exposure in patients with the antisynthetase syndrome. Clin Rheumatol. (2014) 33:221–5. doi: 10.1007/s10067-013-2467-0, PMID: 24384826

[30] Adegunsoye A Oldham JM Demchuk C Montner S Vij R Strek ME. Predictors of survival in coexistent hypersensitivity pneumonitis with autoimmune features. Respir Med. (2016) 114:53–60. doi: 10.1016/j.rmed.2016.03.012, PMID: 27109811 PMC4845760

[31] Fischer A Antoniou KM Brown KK Cadranel J Corte TJ du Bois RM., “ERS/ATS Task Force on Undifferentiated Forms of CTD-ILD” An official European Respiratory Society/American Thoracic Society research statement: interstitial pneumonia with autoimmune features. Eur Respir J (2015); 46: 976–987, doi: 10.1183/13993003.00150-2015, PMID: 26160873

[32] Tzilas V Tzouvelekis A Bouros E Karampitsakos T Ntasiou M Katsaras M. Diagnostic value of BAL lymphocytosis in patients with indeterminate for usual interstitial pneumonia imaging pattern. Eur Respir J. (2019) 54:1901144. doi: 10.1183/13993003.01144-2019, PMID: 31320457

[33] Tzilas V Digalaki A Bouros E Avdoula E Tzouvelekis A Bouros D. Diagnostic utility of Bronchoalveolar lavage lymphocytosis in patients with interstitial lung diseases. Respiration. (2023) 102:944–7. doi: 10.1159/000534429, PMID: 37866357

[34] Chartrand S Swigris JJ Peykova L Chung J Fischer A. A multidisciplinary evaluation helps identify the Antisynthetase syndrome in patients presenting as idiopathic interstitial pneumonia. J Rheumatol. (2016) 43:887–92. doi: 10.3899/jrheum.150966, PMID: 26932342

[35] Barratt SL Adamali HH Cotton C Mulhearn B Iftikhar H Pauling JD. Clinicoserological features of antisynthetase syndrome (ASyS)-associated interstitial lung disease presenting to respiratory services: comparison with idiopathic pulmonary fibrosis and ASyS diagnosed in rheumatology services. BMJ Open Respir Res. (2021) 8:e000829. doi: 10.1136/bmjresp-2020-000829 PMC7798409 33419741

[36] Cavagna L Trallero-Araguás E Meloni F Cavazzana I Rojas-Serrano J Feist E. Influence of Antisynthetase antibodies specificities on Antisynthetase syndrome clinical Spectrum time course. J Clin Med. (2019) 8:2013. doi: 10.3390/jcm8112013, PMID: 31752231 PMC6912490

[37] Raghu G Remy-Jardin M Ryerson CJ Myers JL Kreuter M Vasakova M. Diagnosis of hypersensitivity pneumonitis in adults. An official ATS/JRS/ALAT clinical practice guideline. Am J Respir Crit Care Med. (2020) 202:e36–69. doi: 10.1164/rccm.202005-2032ST, PMID: 32706311 PMC7397797

[38] Churg A Wright JL Ryerson CJ. Pathologic separation of chronic hypersensitivity pneumonitis from fibrotic connective tissue disease-associated interstitial lung disease. Am J Surg Pathol. (2017) 41:1403–9. doi: 10.1097/PAS.0000000000000885, PMID: 28614213

